# The Diagnosis and Treatment of Eczema

**Published:** 1888-09

**Authors:** 


*The Diagnosis and Treatment of Eczema*. By TOM ROBINSON, M.D., Physician to St. John's Hospital for Diseases of the Skin. London: J. & A. CHURCHILL. 1887.

While in certain departments of Medicine (as, for instance, nervous diseases) classification has, during recent years, been continuously undergoing evolution, in that of skin-diseases the opposite process of devolution has markedly been taking place; and instead of the elaborate nomenclature of Willan and Bateman, a much less complex subdivision now obtains. This simplicity is forcibly impressed upon one by the perusal of Dr. Tom Robinson's treatise on Eczema, upon which simplicity practitioners and students are alike to be congratulated. Dr. Robinson's book, besides being free from undue elaboration of nomenclature, is easily intelligible, comparatively brief, and devoid of affectation. If its matter is neither very profound nor very novel, it is thoroughly practical; the section on treatment is entirely worthy of attention, and contains several excellent recipes—always of value in the treatment of a disease so capricious, and often so refractory, as eczema.

